# Left ventricular changes in moderate aortic stenosis in women compared to men

**DOI:** 10.1093/ehjimp/qyag064

**Published:** 2026-04-08

**Authors:** Kyriakos Panaou, Constantijn S Venema, Kees H van Bergeijk, Demetra Hadjicharalambous, Jan A Krikken, Hindrik W van der Werf, Ad F M van den Heuvel, Yvonne L Douglas, Erik Lipsic, Adriaan A Voors, Joanna J Wykrzykowska

**Affiliations:** Department of Cardiology, University Medical Center Groningen, Hanzeplein 1, Groningen, 9713 GZ, The Netherlands; Department of Cardiology, University Medical Center Groningen, Hanzeplein 1, Groningen, 9713 GZ, The Netherlands; Department of Cardiology, University Medical Center Groningen, Hanzeplein 1, Groningen, 9713 GZ, The Netherlands; Department of Cardiology, University Medical Center Groningen, Hanzeplein 1, Groningen, 9713 GZ, The Netherlands; Department of Cardiology, University Medical Center Groningen, Hanzeplein 1, Groningen, 9713 GZ, The Netherlands; Department of Cardiology, University Medical Center Groningen, Hanzeplein 1, Groningen, 9713 GZ, The Netherlands; Department of Cardiology, University Medical Center Groningen, Hanzeplein 1, Groningen, 9713 GZ, The Netherlands; Department of Cardiothoracic Surgery, University Medical Center Groningen, Hanzeplein 1, Groningen, 9713 GZ, The Netherlands; Department of Cardiology, University Medical Center Groningen, Hanzeplein 1, Groningen, 9713 GZ, The Netherlands; Department of Cardiology, University Medical Center Groningen, Hanzeplein 1, Groningen, 9713 GZ, The Netherlands; Department of Cardiology, University Medical Center Groningen, Hanzeplein 1, Groningen, 9713 GZ, The Netherlands

**Keywords:** sex differences, echocardiography, remodelling, concentric hypertrophy, diastolic dysfunction, dyspnoea

## Abstract

**Aims:**

Sex differences in myocardial changes have been identified, but longitudinal investigations in moderate aortic stenosis populations are lacking. The objective of this study was to investigate sex differences in myocardial changes in aortic stenosis.

**Methods and results:**

We retrospectively collected longitudinal echocardiographic and clinical data of 542 patients with a diagnosis of asymptomatic moderate aortic stenosis. Baseline was defined as the first echocardiogram showing non-severe aortic stenosis.

We enrolled 205 (37.8%) females and 337 (62.2%) males, with a median age of 69 years and a median follow-up duration of 6.47 years.

Over the course of aortic stenosis, women had higher left ventricular ejection fraction, lower left ventricular mass index, larger relative wall thickness, and more diastolic dysfunction compared to men. Although the prevalence and incidence of concentric hypertrophy did not differ by sex, women developed concentric hypertrophy and diastolic dysfunction at lower mean gradients than men.

Incidence and time of symptom occurrence did not differ by sex. Overall, 99 (48%) women and 148 (44%) men developed symptoms, at a median age of 73.4 [64.2;81.0] years and a mean gradient of 39.8 [31.5;47.5] mmHg, which was similar between the sexes. When symptomatic, women more commonly presented with dyspnoea (87.8% vs. 74.8%; *P* = 0.021). Incidence and time from baseline to aortic valve replacement and mortality were similar between men and women.

**Conclusion:**

Women with aortic stenosis have worse diastolic function, develop concentric hypertrophy and diastolic dysfunction at lower mean gradients, and more often present with dyspnoea. We observed no sex differences in time from baseline to replacement or mortality.

**Social Media Summary:**

Women with moderate aortic stenosis developed concentric hypertrophy at lower mean gradients, had more and earlier diastolic dysfunction, and more often presented with dyspnoea compared to men. No difference was observed in outcomes. #vhdAS #TAVR #WomenInCardiology

## Graphical Abstract

**Graphical Abstract:**
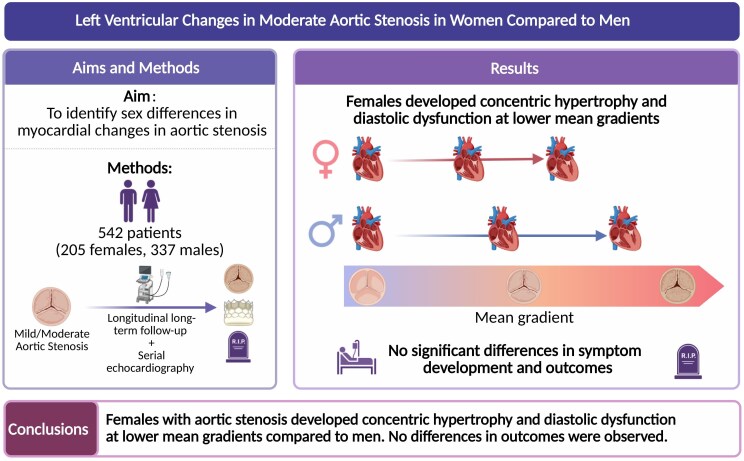

## Introduction

Aortic stenosis (AS) has long been recognized as one of the most common valvular lesions affecting patients older than 65 years.^1,2^ As the global population ages, its prevalence is expected to further increase, with an increasing percentage of women due to their higher life expectancy.^3–7^ Despite this, important gaps in knowledge remain regarding sex differences in the progression of aortic stenosis.

Previous studies have shown conflicting results with regard to progression, cardiac damage, and adverse outcomes between men and women with aortic stenosis. This could be attributed to the wide variation in baseline characteristics and clinical presentation between these studies.^8–13^ Recent investigations on sex differences in patients with aortic stenosis have mainly focused on the development of left ventricular diastolic dysfunction and hypertrophy, which have been associated with severe symptoms and adverse outcomes.^14–16^ While early studies demonstrated a higher prevalence of concentric hypertrophy in women,^17–19^ this could not be confirmed in more recent investigations.^20–23^ A major limitation of these studies is that the majority were cross-sectional investigations, while longitudinal studies are largely missing. Specifically, differences between men and women in the relationship between features of extravalvular adaptation and the severity of aortic stenosis are particularly under-investigated. Given the aforementioned prognostic significance of features such as concentric hypertrophy and diastolic dysfunction, knowledge of such potential differences could improve patient-tailored treatment, particularly regarding the timing of aortic valve replacement (AVR).

In the present study, we aimed to longitudinally compare the progression of aortic stenosis and left ventricular changes between women and men.

## Methods

### Study design

#### Patient population

We conducted a retrospective longitudinal cohort study using data collected from the electronic health records of the University Medical Center Groningen (UMCG), a tertiary referral centre in the Netherlands. We identified patients presenting to the UMCG between December 2011 and December 2022 with moderate AS, defined as aortic valve area (AVA) measured by continuity equation > 1.0 and ≤ 1.5 cm^2^, peak jet velocity ≥ 3.0 and < 4.0 m/s, and mean transvalvular gradient (MG) ≥ 20 and < 40 mmHg. Only patients with at least two recorded echocardiograms, at least 6 months apart (to sufficiently assess progression), which were conducted before AVR (if applicable), were included. Exclusion criteria included prior AV intervention (replacement, repair, or balloon valvuloplasty), subvalvular aortic stenosis, and congenital heart disease apart from bicuspid AV (Supplement  *S1*). Approval was granted by the Medical Ethical Testing Commission of the UMCG, and the requirement for informed consent was waived (METc 2022/545). Baseline was defined as the first echocardiogram showing mild or moderate AS.

### Data collection

#### Clinical data

The standard patient chronological trajectory is displayed in Supplement  *S2*. Demographic variables, medical history, and routine laboratory values were collected within 1 year of baseline echocardiography. Sex assigned at birth was used to classify patients as male or female. Furthermore, clinical outcome variables, including symptom development, AVR (both surgical and transcatheter), all-cause and cardiovascular mortality, hospitalization for heart failure, and myocardial infarction, were recorded until the end of follow-up (December 2022).

#### Echocardiography

Data from all available echocardiograms between December 2011 and December 2022, conducted at the discretion of the treating physician, were collected from each patient from the hospital’s electronic records. Echocardiographic measurements were extracted from clinical reports. Echocardiograms conducted before December 2011 were excluded to ensure adequate image quality and reduce variability in reporting techniques. Baseline was defined as the first echocardiogram to show at least mild AS, defined as peak jet velocity ≥2.6 m/s. Echocardiograms without AS were coded with a negative time from the baseline echocardiogram.

To identify structural and functional differences between men and women at the time of symptom onset and AVR, we identified the closest echocardiogram after symptom onset in patients who became symptomatic and prior to intervention in patients who underwent AVR. These echocardiograms were only considered the peri-symptomatic and peri-procedural echocardiograms if they were conducted within one year of symptom onset or AVR.

For each echocardiogram, valvular parameters (e.g. AVA, peak velocity, and MG), left ventricular ejection fraction (LVEF), left ventricular (LV) dimensions and mass, diastolic parameters (e.g. e’ and E/e’), left atrial volume, right ventricular function and pressure, and the heart rhythm at measurement were collected. LVEF was measured with the Simpson biplane method, and in cases where biplane measurements were not available, eyeballing estimates were collected. Measurements such as left ventricular mass index (LVMI), relative wall thickness (RWT), stroke volume index (SVi), and AVA indexed to body surface area (AVAi), were calculated using the relevant parameters based on consensus definitions for 2D echocardiography. Diastolic dysfunction was defined based on the American Society of Echocardiography diagnostic criteria,^24^ where less than two of the relevant parameters were missing.

The median left ventricular outflow tract (LVOT) diameter was calculated for each patient and was subsequently used to calculate AVA using the continuity equation with velocity time integral ratio, in order to reduce interobserver variability. Peak jet velocity ratio was used to determine AVA if the velocity time integral ratio was not available.

To compare rates of aortic stenosis and geometric progression, the annual change in AV parameters (peak jet velocity, AVA, and mean pressure gradient) and structural and functional parameters (LVMI, RWT, and e’ average) was calculated for each patient using an ordinary least squares regression model to the measurements over time in years. The slope of the resulting regression lines was then used as the annual progression rate. The estimated associations between LVMI, RWT, and E/e’ with mean gradient were plotted for males and females as trajectories using restricted cubic splines with mixed effects models to account for the effects of repeated measurements for each patient.

Left ventricular geometric patterns were defined with sex-specific cutoffs according to current guidelines.^25^ Concentric hypertrophy was defined as LVMI > 95 g/m^2^ in women and > 115 g/m^2^ in men, with relative wall thickness (RWT) > 0.42. Eccentric hypertrophy was defined as LVMI > 95 g/m^2^ in women and > 115 g/m^2^ in men, with RWT ≤ 0.42. Concentric remodelling was defined as LVMI ≤ 95 g/m^2^ in women and ≤ 115 g/m^2^ in men, with RWT > 0.42. These geometric patterns were classified and analysed at baseline and at the echocardiogram closest to 3 years after baseline (with a 1-year window). The prevalence of each remodelling phenotype was compared from baseline to three year follow-up descriptively, only in patients who had recorded values for LVMI and RWT at both baseline and follow-up to ensure comparison of the same patient population across the same follow-up duration.

### Statistical analysis

All statistical analyses were conducted using R version 4.4.0 (R Core Team, 2024). Continuous variables are presented as mean ± SD for normally distributed variables or median (IQR) for non-normally distributed variables. Categorical variables are presented as numbers (percentages). Baseline clinical and echocardiographic characteristics were compared between men and women, using two-sample t-tests for normally distributed variables, Wilcoxon rank-sum tests for non-normally distributed variables, and Pearson χ^2^ or Fisher’s exact tests for categorical data. Two-sided *P* values <0.05 were considered statistically significant.

Time from baseline to symptom development, AVR, and mortality was compared between men and women using univariate Cox proportional hazard models and Kaplan-Meier curves. The relationship of concentric hypertrophy and diastolic dysfunction development and mean pressure gradient was assessed with cumulative incidence curves.

To test the robustness of our findings, we conducted several sensitivity analyses. Echocardiographic measures and clinical endpoints were assessed in a subset excluding patients with reduced ejection fraction or stroke volume index or with a history of coronary artery disease or chest irradiation. Analysis of echocardiographic parameters and Kaplan-Meier analysis for symptom development, AVR, and mortality were repeated using a baseline of the first echocardiogram showing moderate AS (excluding echocardiograms with mild severity). Furthermore, to assess the robustness of the associations between sex and concentric hypertrophy and diastolic dysfunction, we performed Cox proportional hazards event-history analyses along a continuous exposure axis of mean gradient, adjusting for baseline hypertension and coronary artery disease. Total population and missing values for each table can be found in Supplements  *S7*  to  *S10*.

## Results

### Baseline characteristics

We identified 686 patients with moderate aortic stenosis and at least two echocardiograms between December 2011 and December 2022. Of these, 144 patients were subsequently excluded due to prior intervention (n = 58), insufficient echocardiograms (n = 54), echocardiograms performed less than 6 months apart (n = 16), or subvalvular aortic stenosis or congenital heart disease (n = 16) (Supplement  *S1*). Of the 542 included patients, 205 (37.8%) were female, with a median age at baseline of 69 years (*Table 1).* Age and follow-up duration were comparable in both sexes (6.83 vs. 6.34 years, *P* = 0.551).

**Table 1:** Baseline characteristics

	Female (N = 205)	Male (N = 337)	*P*
Total follow-up duration	6.83 [4.08;8.76]	6.34 [4.11;8.99]	0.551
Age at diagnosis	70.0 [62.0;78.0]	68.0 [59.0;75.0]	0.112
Current smoker	61 (29.8%)	110 (32.6%)	0.545
BMI (kg/m^2^)	27.1 [23.6;30.9]	27.2 [24.1;30.2]	0.776
Bicuspid aortic valve	34 (16.6%)	58 (17.2%)	0.944
Diabetes	49 (23.9%)	72 (21.4%)	0.561
Hypertension	124 (60.5%)	200 (59.3%)	0.863
Coronary artery disease	23 (11.2%)	97 (28.8%)	< 0.001
Previous myocardial infarction	11 (5.37%)	46 (13.6%)	0.004
Atrial fibrillation	35 (17.1%)	66 (19.6%)	0.539
Heart failure	13 (6.34%)	27 (8.01%)	0.581
Intracardiac device	12 (5.85%)	17 (5.04%)	0.834
Previous CVA	25 (12.2%)	52 (15.4%)	0.358
Peripheral vascular disease	13 (6.34%)	40 (11.9%)	0.051
Chronic kidney disease (eGFR < 60)	16 (7.80%)	37 (11.0%)	0.290
Chronic dialysis	10 (4.88%)	22 (6.53%)	0.547
Chest irradiation	16 (7.80%)	12 (3.56%)	0.049
COPD/asthma	38 (18.5%)	44 (13.1%)	0.109
Beta-blocker	82 (40.0%)	150 (44.5%)	0.347
ACE-inhibitors	61 (29.8%)	110 (32.6%)	0.545
MRA	4 (1.95%)	20 (5.93%)	0.049
Statin	80 (39.0%)	165 (49.0%)	0.030
Oral anticoagulant	39 (19.0%)	60 (17.8%)	0.809
NT-proBNP (pg/mL)	314 [168;758]	337 [141;818]	0.782
Hemoglobin (mmol/L)	8.00 [7.50;8.60]	8.70 [7.70;9.50]	< 0.001
Sodium (mmol/L)	140 [138;142]	140 [138;142]	0.338
Potassium (mmol/L)	4.20 [3.98;4.40]	4.30 [4.00;4.53]	0.020
BUN (mg/dL)	16.8 [13.7;21.0]	18.8 [14.8;24.4]	0.002
Creatinine (umol/L)	69.0 [59.0;82.0]	92.0 [77.0;113]	< 0.001
eGFR (CKD-EPI)	78.0 [60.5;90.0]	73.0 [57.0;88.2]	0.248
Total cholesterol	4.90 [4.20;5.80]	4.20 [3.50;5.10]	< 0.001
LDL-cholesterol	3.00 [2.25;3.75]	2.40 [1.90;3.30]	0.001
HDL-cholesterol	1.50 [1.20;1.80]	1.10 [0.90;1.42]	< 0.001
Triglycerides	1.43 [1.02;2.06]	1.65 [1.13;2.28]	0.049

BMI = Body Mass Index, CVA = Cerebrovascular accident, eGFR = estimated Glomerular Filtration Rate, COPD = Chronic Obstructive Pulmonary Disease, ACE = angiotensin-converting enzyme, MRA = Mineralocorticoid Receptor Antagonist, NT-proBNP = N-terminal pro-B-type natriuretic peptide, BUN = Blood Urea Nitrogen, LDL = Low-Density Lipoprotein, HDL = High-Density Lipoprotein.

Most comorbidities, including diabetes, hypertension, pre-existing heart failure, and atrial fibrillation, were consistent between sexes. Women had less coronary artery disease and history of myocardial infarction, took fewer MRAs and statins, and had lower blood urea nitrogen and creatinine, with similar eGFR (*Table 1*). All types of cholesterol were higher in women, while triglycerides were lower.

### Baseline echocardiography

At baseline (first echocardiogram showing at least mild or moderate AS), 80.8% of patients had moderate AS, with no difference between the sexes (*Table 2, P* = 0.105). Women had a smaller unindexed AVA, a slightly larger indexed AVA, and a slightly lower peak aortic jet velocity, but with a similar mean pressure gradient.

**Table 2:** Baseline echocardiographic characteristics

	Female (N = 205)	Male (N = 337)	*P*
Baseline AS severity:			0.105
Mild	27 (13.2%)	42 (12.5%)	
Moderate	159 (77.6%)	279 (82.8%)	
Severe	19 (9.27%)	16 (4.75%)	
AVAc	1.21 [1.09;1.41]	1.32 [1.16;1.50]	< 0.001
AVAc indexed to BSA	0.67 [0.60;0.78]	0.65 [0.56;0.74]	0.022
Peak jet velocity	3.00 [2.70;3.20]	3.10 [2.80;3.40]	0.002
Mean gradient	20.9 [17.0;24.2]	21.4 [17.6;26.8]	0.076
LVEF < 50%	10 (4.88%)	37 (11.0%)	0.022
SV index < 35 mL/m^2^	24 (13.6%)	47 (15.3%)	0.696
EF/Flow category:			0.103
Low EF, low-flow	3 (1.69%)	11 (3.58%)	
Low EF, normal-flow	5 (2.82%)	23 (7.49%)	
Normal EF, low-flow	21 (11.9%)	36 (11.7%)	
Normal EF, normal-flow	148 (83.6%)	237 (77.2%)	
RWT	0.48 [0.39;0.55]	0.44 [0.39;0.53]	0.038
LVMI	83.5 [66.0;96.0]	96.0 [79.0;113]	< 0.001
Left Ventricular Hypertrophy (LVH)	50 (26.9%)	71 (23.0%)	0.384
e’ average	7.65 [5.90;9.67]	8.10 [7.00;10.2]	0.005
E/e’	10.4 [8.30;13.6]	8.90 [7.20;11.6]	< 0.001
E/A	0.82 [0.67;1.02]	0.87 [0.71;1.10]	0.048
Diastolic Dysfunction (ASE Definition)	68 (49.6%)	86 (36.6%)	0.019
Pulmonary Artery Systolic Pressure	33.3 [28.4;40.4]	32.4 [27.8;37.4]	0.290
Tricuspid Regurgitation Peak Velocity	2.70 [2.40;3.00]	2.60 [2.40;2.80]	0.257
Mitral Regurgitation:			0.358
None-Trace	144 (71.3%)	261 (77.4%)	
Mild	46 (22.8%)	57 (16.9%)	
Moderate	11 (5.45%)	17 (5.04%)	
Severe	1 (0.50%)	2 (0.59%)	
Aortic Regurgitation:			0.209
None-Trace	142 (70.3%)	210 (62.3%)	
Mild	45 (22.3%)	94 (27.9%)	
Moderate	13 (6.44%)	31 (9.20%)	
Severe	2 (0.99%)	2 (0.59%)	
Tricuspid Regurgitation:			0.006
None-Trace	138 (68.7%)	267 (79.9%)	
Mild	47 (23.4%)	57 (17.1%)	
Moderate	12 (5.97%)	9 (2.69%)	
Severe	4 (1.99%)	1 (0.30%)	

AS = aortic stenosis, AVA = aortic valve area, BSA = body surface area, LVEF = left ventricular ejection fraction, SV = stroke volume, EF = ejection fraction, RWT = relative wall thickness, LVMI = left ventricular mass index.

Men more commonly had an LVEF of <50%. Women had smaller LV end diastolic and systolic dimensions, and smaller interventricular septum and posterior wall diameters. Despite having a higher RWT (0.48 vs. 0.44, *P* = 0.038) and a lower LVMI (83.5 vs. 96.0, *P* < 0.001), the incidence of LV hypertrophy was similar (26.9% vs. 23.0%, *P* = 0.384) between sexes. Most patients (80%) had a normal EF, normal-flow status, which did not differ by sex. Women also exhibited more diastolic dysfunction (49.6% vs. 36.6%, *P* = 0.019), as indicated by a lower e’ average (7.65 vs. 8.10, *P* = 0.005), higher E/e’ (10.4 vs. 8.9, *P* < 0.001), and lower E/A (0.82 vs. 0.87, *P* = 0.048). Additionally, tricuspid regurgitation was more common in women, although all other relevant echocardiographic parameters did not show significant sex-based differences (*Table 2*).

The distribution of LV geometry patterns at baseline (*Table 3*) showed no significant sex-based variation. The most common pattern was normal geometry in both men and women (38.9%), closely followed by concentric remodelling (37.3%). Concentric hypertrophy had an overall baseline prevalence of 18.4%, being slightly more common in women (20.4% vs. 17.1%, *P* = 0.631), although not statistically significant.

**Table 3:** Patterns of LV geometry at baseline and 3-year follow-up

	*Baseline Echocardiography*				*Three-Year Follow-Up*			
	All (N = 244)	Female (N = 98)	Male (N = 146)	*P*	All (N = 244)	Female (N = 98)	Male (N = 146)	*P*
Concentric Remodelling	91 (37.3%)	36 (36.7%)	55 (37.7%)	0.989	107 (43.9%)	40 (40.8%)	67 (45.9%)	0.515
Concentric Hypertrophy	45 (18.4%)	20 (20.4%)	25 (17.1%)	0.631	69 (28.3%)	29 (29.6%)	40 (27.4%)	0.820
Eccentric Hypertrophy	13 (5.33%)	6 (6.12%)	7 (4.79%)	0.871	19 (7.79%)	7 (7.14%)	12 (8.22%)	0.949
Normal Geometry	95 (38.9%)	36 (36.7%)	59 (40.4%)	0.657	49 (20.1%)	22 (22.4%)	27 (18.5%)	0.553

### Echocardiographic changes from baseline to last follow-up

#### Rates of change

Patients received a median of five follow-up echocardiograms, which was not different between sexes. Regression-derived annual rates of change (slopes) of AS-related and LV-related structural and functional parameters are shown in *Table 4*. Slopes of measures of AS severity, including AVA, mean gradient, and peak jet velocity, did not differ between men and women. However, men had a significantly faster annual rate of increase in LVMI compared to women (3.2 vs. 2.0 g/m^2^/year, *P* = 0.033).

**Table 4:** Rates of change of echocardiographic parameters

	Female (N = 205)	Male (N = 337)	*P*
AVA Slope (change per year)	−0.08 [−0.14;−0.03]	−0.09 [−0.15;−0.04]	0.097
Mean Gradient Slope (change per year)	2.91 [1.29;5.98]	3.26 [1.65;5.66]	0.794
Peak Velocity Slope (change per year)	0.19 [0.08;0.31]	0.17 [0.09;0.29]	0.799
Rapid progression	99 (48.8%)	171 (50.7%)	0.722
LVMI Slope (g/m^2^/year)	1.98 [−0.17;5.38]	3.20 [−0.13;8.08]	0.033
RWT Slope	0.00 [−0.02;0.02]	0.01 [−0.01;0.02]	0.327
e’ Average Slope	−0.12 [−0.43;0.13]	−0.19 [−0.50;0.09]	0.326

AVA = aortic valve area, LVMI = left ventricular mass index, RWT = relative wall thickness.

#### LV structural and functional patterns throughout follow-up

**Figure 1:**
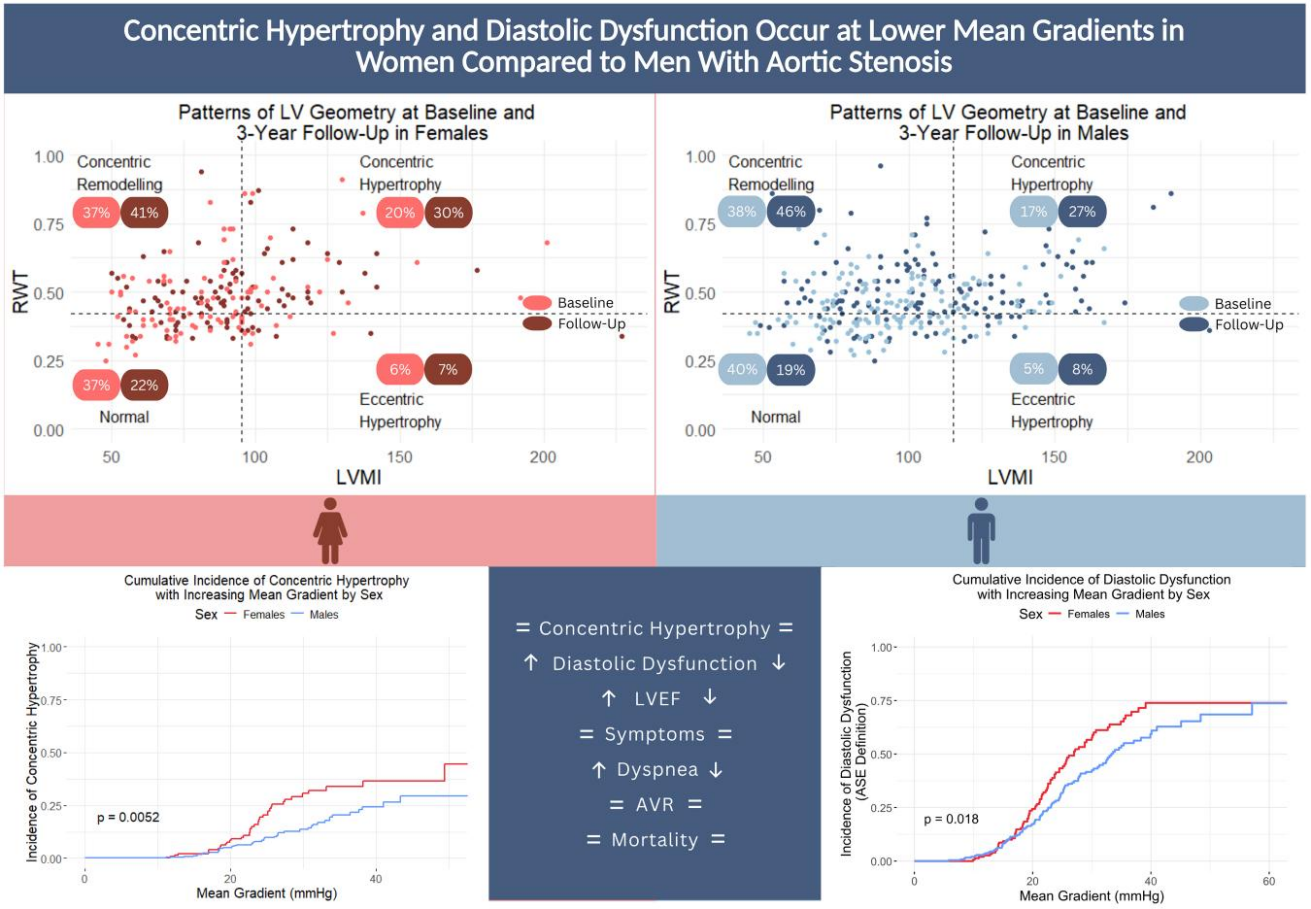
The top panels display scatterplots of the left ventricular (LV) geometry phenotypes, as the relationship between left ventricular mass index (LVMI) and relative wall thickness (RWT). These show similar prevalence and progression. On the bottom left is a cumulative incidence graph showing that women develop concentric hypertrophy at a lower mean gradient. On the bottom right is a cumulative incidence graph showing that women develop diastolic dysfunction at a lower mean gradient. LVEF = Left Ventricular Ejection Fraction; AVR = Aortic Valve Replacement

No significant sex-based differences were found in the prevalence of concentric remodelling, concentric hypertrophy, or eccentric hypertrophy at any measurement (*Table 3* and *Figure 1*). In total, 244 patients had recorded values for LVMI and RWT both at baseline and at the echocardiogram closest to 3 years from baseline. At the 3-year follow-up, normal geometry decreased from 38.9% to 20.1%, with similar trends in both sexes. Concentric hypertrophy increased from 18.4% to 28.3%, concentric remodelling from 37.3% to 43.9%, and eccentric hypertrophy from 5.3% to 7.8%, all showing similar patterns between men and women.

**Figure 2:**
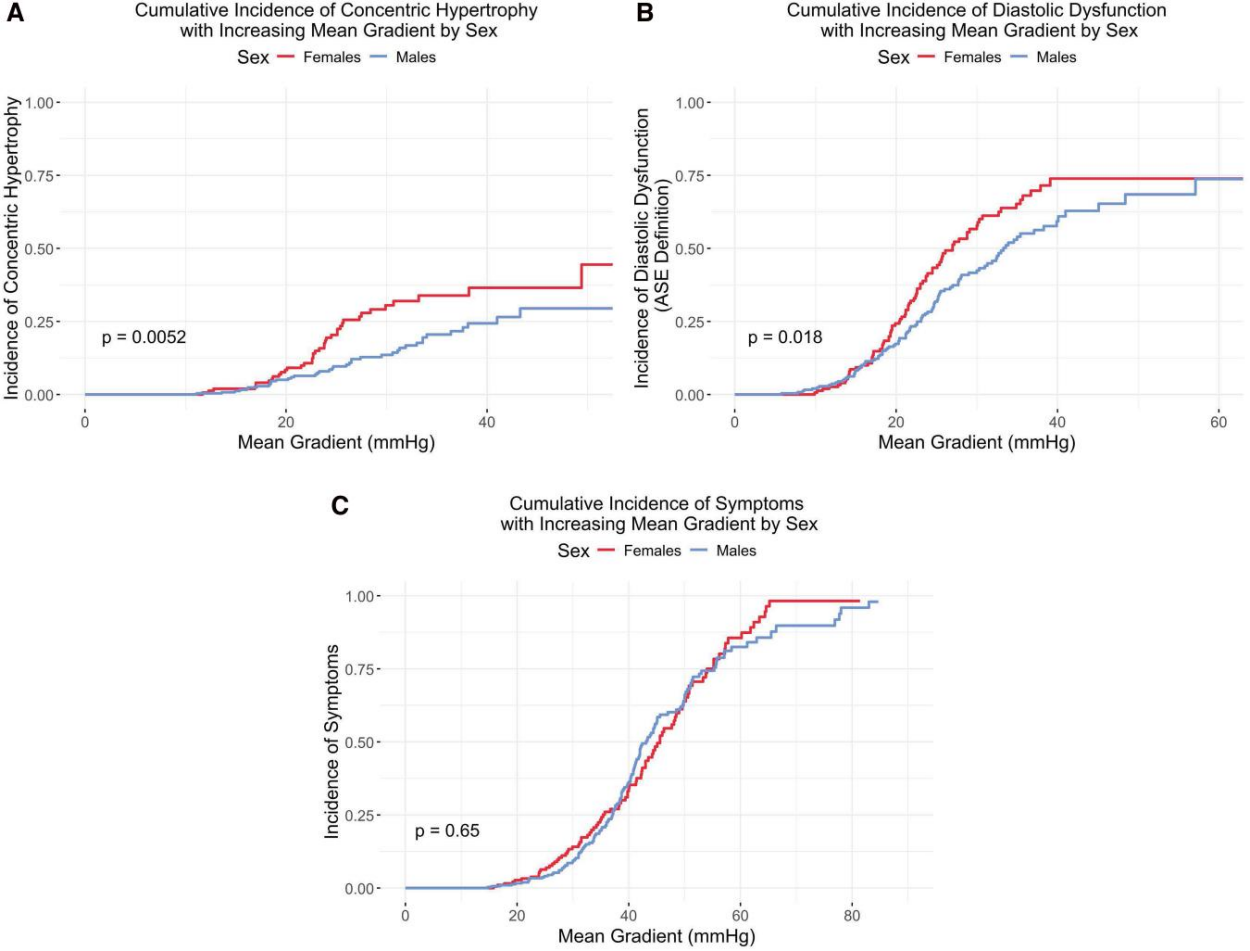
Cumulative incidence of concentric hypertrophy, diastolic dysfunction, and symptoms by mean gradient. *A*) Cumulative incidence curve of concentric hypertrophy by increasing mean gradient. The female curve is shifter to the left, using the first echocardiogram to show concentric hypertrophy in each patient, suggesting concentric hypertrophy at lower mean gradients. *B*) Cumulative incidence graph of diastolic dysfunction by increasing mean gradient. The female curve is shifted to the left, suggesting diastolic dysfunction at lower mean gradients. *C*) Cumulative incidence curve of symptoms by increasing mean gradient, showing no significant sex difference. (n = 542 patients).

*Figure 2A* shows the cumulative incidence of concentric hypertrophy with increasing mean gradient by sex. The curve for women shows a significant shift towards lower gradients compared to men (*P* = 0.005), indicating a higher probability of concentric hypertrophy at lower mean gradients in women. The mean gradient at the first echocardiogram showing concentric hypertrophy was also significantly lower in women compared to men (24.5 vs. 29.7 mmHg, *P* = 0.007).

A similar pattern was observed for diastolic dysfunction, as seen in *Figure 2B*. The cumulative incidence curve of diastolic dysfunction with increasing mean gradient was also significantly shifted to the left in women (*P* = 0.018), suggesting that diastolic dysfunction develops at lower pressure gradients in women. The relationship of mean gradient with LVMI, RWT, and E/e’ can be seen in Supplement  *S3*.

#### Echocardiographic findings at symptom development and AVR (*S*upplement  *S4* and *S5*)

The echocardiogram closest to symptom onset and AVR was performed a median of 3.8 years from baseline and was similar between sexes. At both symptom onset and AVR, AVA indexed to body surface area, peak jet velocity, and mean gradient were similar between the sexes. The last available echocardiogram showed similar trends (Supplement  *S6)*.

#### Symptom development

**Figure 3:**
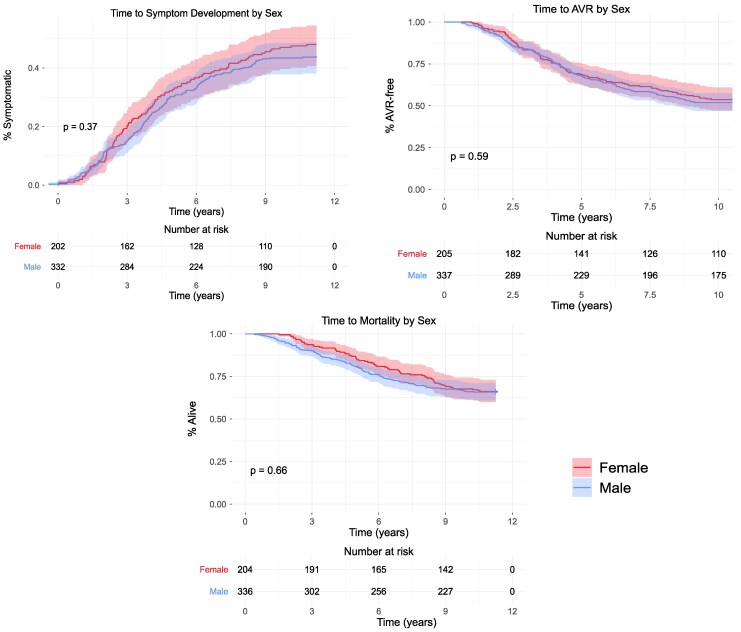
Kaplan-Meier curves for time to symptoms, AVR, and mortality. Time-to-event curves for symptom development, aortic valve replacement (AVR), and mortality, stratified by sex. These demonstrate a similar overall incidence and time to event for all three outcomes between men and women. (n = 542 patients).

*Figure 2C* shows that symptom onset occurred at the same mean aortic valve pressure gradient in men and women. A similar proportion of men (44%) and women (48%) developed symptoms of aortic stenosis by the end of follow-up (*P* = 0.30), with a comparable time to symptom development of 3.8 years (*P* = 0.370) (*Figure 3*).

**Figure 4:**
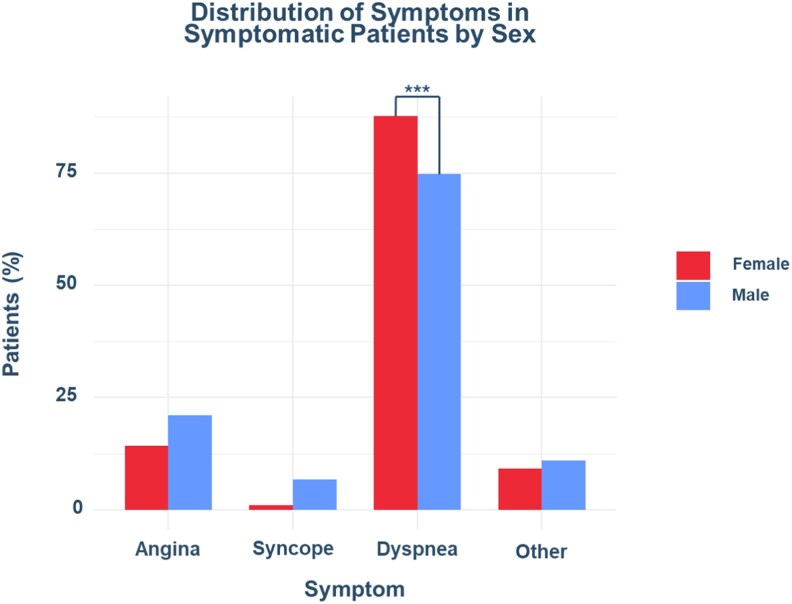
Distribution of symptoms in symptomatic patients. Bar graph showing the percentage of symptomatic patients that developed each symptom (some patients developed more than one). Dyspnoea was the most common and was significantly more common in women compared to men. (n = 542 patients).

Dyspnoea was the most common symptom (36% overall, 80% in symptomatic patients), followed by angina (8.3% overall, 18.4% in symptomatic patients). Dyspnoea was more common in women than men (87.8% vs. 74.8%, *P* = 0.021). Other symptoms were not different between men and women (*Figure 4*).

#### Clinical outcomes

Overall, 47.8% of patients underwent AVR, at a median time of 3.96 years. The incidence and time to aortic valve replacement were not significantly different between men and women (*Figure 3*). Of the symptomatic patients, 82.7% of symptomatic women and 82.3% of symptomatic men underwent AVR. The type of replacement was also not significantly different between men and women, 39% of replacements being transcatheter, and 61% being surgical.

Overall, 34.3% of patients died during follow-up. No sex-dependent difference was observed in the incidence, time, or cause of death (*Figure 3*).

Rates of adverse events, including heart failure hospitalizations, new-onset heart failure, and new-onset atrial fibrillation, were comparable between men and women (*Table 5*). Coronary artery bypass graft surgery was more common in males.

**Table 5:** Outcomes

	Female (N = 205)	Male (N = 337)	*P*
Hospitalization for heart failure	30 (14.6%)	57 (17.1%)	0.523
Number of HF hospitalizations	1.00 [1.00;2.00]	1.00 [1.00;2.00]	0.564
Death	70 (34.1%)	116 (34.4%)	1.000
Cause of death:			0.472
Cardiovascular	16 (23.5%)	28 (24.1%)	
Non-cardiovascular	24 (35.3%)	50 (43.1%)	
Unknown	28 (41.2%)	38 (32.8%)	
New-onset heart failure	25 (13.1%)	37 (12.0%)	0.830
New-onset atrial fibrillation	33 (19.4%)	66 (23.7%)	0.340
Myocardial infarction:			0.687
No	193 (94.1%)	310 (92.0%)	
NSTEMI	8 (3.90%)	19 (5.64%)	
STEMI	4 (1.95%)	8 (2.37%)	
Percutaneous coronary intervention	16 (7.80%)	40 (11.9%)	0.173
CABG	8 (3.90%)	30 (8.90%)	0.042
Cerebrovascular accident	11 (5.39%)	24 (7.19%)	0.523
Peripheral artery disease intervention/surgery	9 (4.79%)	17 (5.35%)	0.947
Device implantation:			0.105
No	162 (92.6%)	254 (90.7%)	
Pacemaker	12 (6.86%)	16 (5.71%)	
ICD	1 (0.57%)	2 (0.71%)	
CRT	0 (0.00%)	8 (2.86%)	

HF = heart failure, NSTEMI = non-ST-elevation myocardial infarction, STEMI = ST-elevation myocardial infarction, CABG = coronary artery bypass graft, ICD = intracardiac cardioverter defibrillator, CRT = cardiac resynchronization therapy

#### Sensitivity analysis

We conducted a sensitivity analysis assessing echocardiographic parameters using a modified baseline of only moderate AS (excluding echocardiograms with mild severity) and excluding patients with a history of coronary artery disease or chest irradiation, reduced EF, or low SVi (Supplement  *S11*). The results were largely consistent with the main analysis. One significant difference is that in this subset, left atrial volume index (LAVI) was significantly higher in women (*P* = 0.030). The graphs of the cumulative incidence of concentric hypertrophy and diastolic dysfunction by increasing mean gradient were also repeated, excluding patients with a reduced EF or SVi, with consistent results (Supplement  *S12*). Females consistently showed concentric hypertrophy at lower mean gradients, remaining significant after adjustment for hypertension and coronary artery disease (HR 0.563, *P* = 0.013). Similarly, the association between female sex and diastolic dysfunction at lower mean gradients not only remained significant but was strengthened after adjustment for hypertension and coronary artery disease (HR 0.64, *P* = 0.003). The results of the Kaplan-Meier analysis for symptom development, AVR, and mortality using a moderate (vs. mild or moderate) baseline were in line with the main findings, showing no significant differences between men and women (Supplement  *S13*).

## Discussion

In a single-centre study in patients with moderate aortic stenosis, we demonstrated that women develop concentric hypertrophy and diastolic dysfunction at lower mean pressure gradients compared to men. In addition, women are more likely to present with dyspnoea. Finally, with early diagnosis and equal follow-up between sexes, no significant differences in adverse outcomes or time to AVR were observed.

### Left ventricular adaptation

While no clinically significant differences in valve-related parameters were observed, we did identify sex-dependent variation in the left ventricular response to aortic stenosis. Specifically, women showed markedly more pronounced diastolic dysfunction, which occurred at lower aortic stenosis severity compared to men. This adds previously missing longitudinal data, which strengthens the results of previous cross-sectional investigations showing a similar trend.^26–29^

This observation may also be related to another key finding of this study, namely the development of concentric hypertrophy at lower mean gradients in women compared to men. To the best of our knowledge, no previous investigation has directly examined sex differences in the relationship between mean pressure gradient and concentric hypertrophy or diastolic dysfunction development.

The mean gradient at the first echocardiography showing concentric hypertrophy was 24.5 mmHg in women and 29.7 mmHg in men, a potentially clinically relevant difference. This suggests that female left ventricles are more sensitive to increased intraventricular pressure, leading to a greater hypertrophic response and diastolic dysfunction. Several studies have associated heart failure with preserved ejection fraction (HFpEF)-like phenotype, characterized by left ventricular hypertrophy and diastolic dysfunction, with more severe symptoms of dyspnoea, and worse long-term outcomes.^14–16^ This offers one potential explanation regarding the higher incidence of dyspnoea in women compared to men in our cohort. Moreover, this indicates that women may benefit more from a medical treatment targeted at extravalvular adaptations compared to men.

Importantly, the overall incidence of new concentric hypertrophy did not differ by the time of last follow-up. A significant consideration, however, is that the mean pressure gradients at the time of both concentric hypertrophy and diastolic dysfunction development fall well within the moderate aortic stenosis range (20–40 mmHg). Therefore, patients appear to develop these changes in a very early stage of disease progression, and it is in this moderate stage that we observe the most significant difference between men and women. This difference diminishes as patients progress such that by the time of AVR, both sexes have the same degree of hypertrophy. Nevertheless, women maintain a greater degree of diastolic dysfunction throughout follow-up, suggesting that the observed differences persist. Given these results and the adverse prognostic implications of diastolic dysfunction and advanced concentric hypertrophy, earlier intervention may be warranted.

### Symptom development

Previous results have suggested that women are more symptomatic at presentation, and specifically tend to report dyspnoea more commonly than men.^7,11,12,22,30^ We did indeed confirm that symptomatic females more commonly present with dyspnoea compared to symptomatic males, although we did not observe a higher overall incidence of symptoms in women. The time from baseline to symptom development was also similar between men and women, suggesting that sex-dependent variation in pathophysiological processes does not cause differences in the timing of symptom onset, as previously postulated. One proposed explanation for the discordant results between our study and previous investigations^7,12,30^ is the longitudinal nature of this study with an asymptomatic mild or moderate AS baseline. This minimized delays between disease onset and first medical contact, a proposed mechanism behind the higher incidence of symptoms in women observed in previous studies.

Nevertheless, the higher incidence of dyspnoea in women in the symptomatic subgroup indicates a difference in the underlying pathophysiology, possibly related to the observed differences in the severity of diastolic dysfunction and the development of concentric hypertrophy at a lower mean gradient.

One disconnect is the absence of a sex-mediated effect in the relationship between mean pressure gradient and symptom development. Given the previously discussed finding of concentric hypertrophy and diastolic dysfunction at lower mean pressure gradients in women, and the link between these features and symptom development in past literature, it follows that women would be more likely to develop symptoms at a lower mean gradient. However, our data did not support this. This may imply yet again some sex-dependent variation in the mechanism through which left ventricular structure and function are associated with symptom development, and its relationship with intraventricular pressure. A possible explanation is that women may have a lower capacity to maintain wall stress under increased intraventricular pressure, leading to a stronger hypertrophic response. This could potentially delay symptom onset, such that symptoms occur at a similar mean gradient to those of men.

### AVR and outcomes

Notably, the frequency and timing of aortic valve replacement did not differ between men and women. Given the similarity in the time to symptom development between men and women, this was an expected finding. This is, albeit, at odds with findings from several preceding studies, which suggest a delay in replacement in women.^12,13^ This may be due to design differences, as our investigation followed these patients from a pre-symptomatic stage.

In our population, the incidence and time from baseline to death were not significantly different between men and women. Furthermore, no major differences were observed in any adverse events, contrasting with previous studies, which showed conflicting results.^7–9,12,13^

Finally, though we recognize the limitations of our design, as described below, the robustness of our results to various sensitivity analyses indicates that residual confounding is unlikely to be of significant effect in the observed associations.

### Clinical implications

Recognizing sex-specific differences in myocardial remodelling is an important step toward defining what constitutes a normal vs. abnormal echocardiographic phenotype in both males and females with moderate aortic stenosis. This may ultimately inform more individualized echocardiographic interpretation and clinical management of aortic stenosis. Whether such individualized care translates into improved outcomes warrants further prospective investigation. Recent results propose aortic valve replacement at an earlier stage than the current guideline-recommended stage.^31–34^ Given these data, identification of high-risk features in patients at an early stage of disease progression would be of high value in AVR-related decision-making. It is in this stage that we identified important sex differences in the development of concentric hypertrophy and diastolic dysfunction. Therefore, sex may be an important consideration when interpreting echocardiographic parameters in the moderate stage, and when taking these high-risk features into account in planning future interventions.

Seeing as signs of LV hypertrophy and myocardial damage were observed at lower mean gradients in women, the introduction of sex-specific cutoffs for echocardiographic severity measures may significantly improve correct severity staging and optimal treatment for women. Further studies are required to determine the mean pressure gradient threshold at which myocardial damage becomes irreversible, and before which treatment aimed at preventing progression of myocardial damage is necessary, either by mechanical afterload reduction (AVR) or medical therapy. Further investigation is also necessary to identify whether the observed sex-specific differences in myocardial changes in moderate AS translate into differences in the likelihood or extent of reverse remodelling after AVR.

## Limitations

This study is limited by several factors. First, data from a single tertiary referral centre may not apply to other populations, especially in less specialized settings, due to differences in patient characteristics and follow-up practices. Furthermore, the retrospective nature of this investigation provides several challenges, including non-standardized follow-up and treatment, reliance on clinically-collected records from medical charts, missing information, and potential interobserver variability in echocardiographic measurements. Non-standardized follow-up at the discretion of the treating physician may have introduced selection bias, with more frequent follow-up with more severe illness. Additionally, the challenges of accurately retrospectively assessing the onset of symptoms may limit the accuracy of relevant results. To reduce the effect of these limiting factors, we included patients with at least one echocardiogram meeting the criteria for moderate aortic stenosis and excluded echocardiograms before December 2011. We identified and selected all patients meeting these requirements using an extensive search protocol in the hospital’s electronic records, thereby reducing selection bias. Still, changes in standard practice and technological advancements during our study period may have impacted results over time. In addition, our results rely solely on echocardiographic data, with no available magnetic resonance imaging, which has higher accuracy for assessing cardiac structure and function. Moreover, further grading of diastolic dysfunction could not be accurately performed due to the lack of some echocardiographic parameters in this clinical cohort. Furthermore, while we carried out sensitivity analyses for some potential confounders, we did not correct for differences in baseline characteristics in our statistical analysis, thereby potentially overlooking some confounding effects. Finally, no information about gender identity or gender-affirming treatment was available, thus limiting interpretation to strictly biological sex at birth.

## Conclusions

Women with aortic stenosis show more pronounced diastolic dysfunction, develop concentric hypertrophy and diastolic dysfunction at lower mean pressure gradients, and more frequently present with dyspnoea. Despite these variations, with early diagnosis and equal follow-up, aortic valve replacement and adverse outcomes did not differ in incidence or timing.

## Supplementary Material

qyag064_Supplementary_Data

## Data Availability

Raw data analyzed and presented in this manuscript are available upon reasonable request to the corresponding author.

## Lead author biography

Kyriakos Panaou was born in Cyprus but emigrated to the United States at the age of nine, where he completed the majority of his primary and secondary education. He completed two years of a Biology degree as a dual enrollment student at the University of Georgia. He subsequently began his medical education at the University of Groningen in 2022, receiving a Bachelor of Science in Medicine in 2025. He has been an active cardiology researcher at the University Medical Center Groningen since 2023, completing several first-author manuscripts on the topic of myocardial changes in aortic stenosis, with a particular interest in sex differences and pathophysiology. He is a co-author on a published international review of aortic stenosis and TAVI in women (*Structural Heart,* 2025), has previously presented his research at the TCT and London Valves conferences, and is involved in the daily operation of ongoing studies. This work, among other investigations, has been completed in preparation for an MD/PhD trajectory at the University of Groningen, under the supervision of dr. Joanna J. Wykrzykowska and Prof. dr. Adriaan A. Voors.
