# Supplementary material for: Left ventricular changes in moderate aortic stenosis in women compared to men
Source: Eur Heart J Imaging Methods Pract. 2026 Apr 8;4(2):qyag064. doi: 10.1093/ehjimp/qyag064 (PMC13289755; doi:10.1093/ehjimp/qyag064)

## Supplement

Supplement 1 - Inclusion Flow Chart


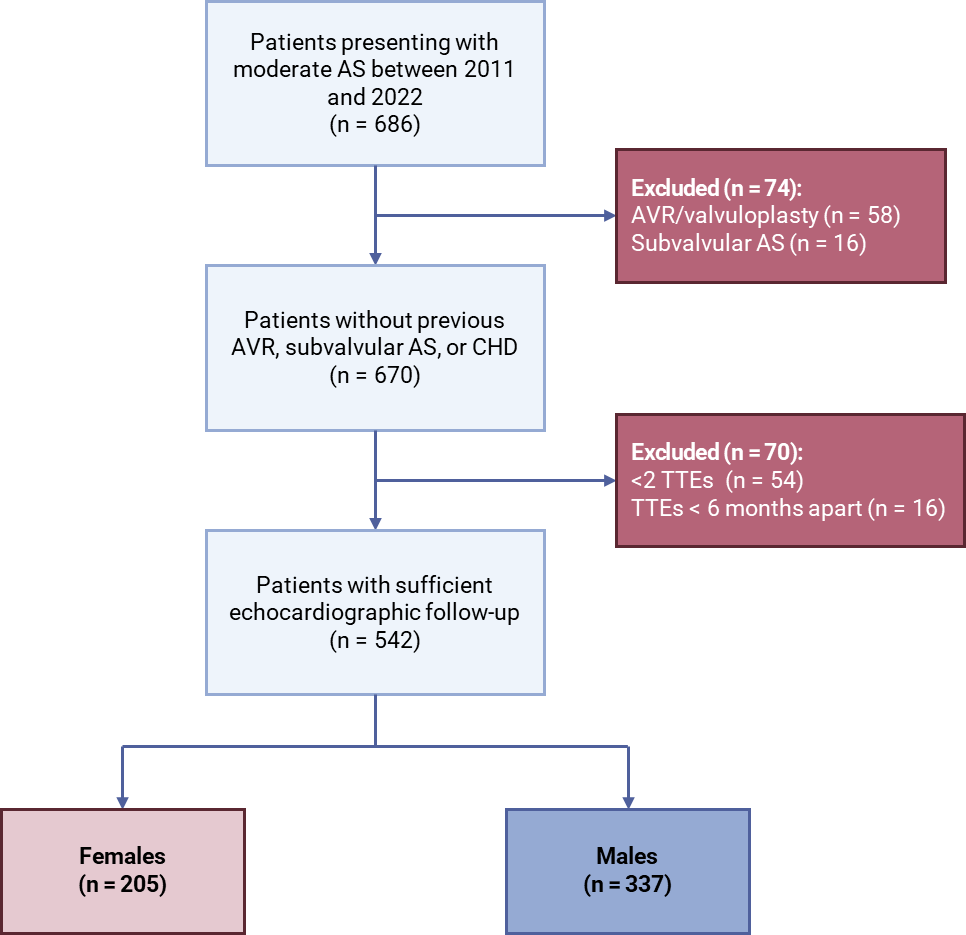


Supplement 2 – Patient Trajectory

###
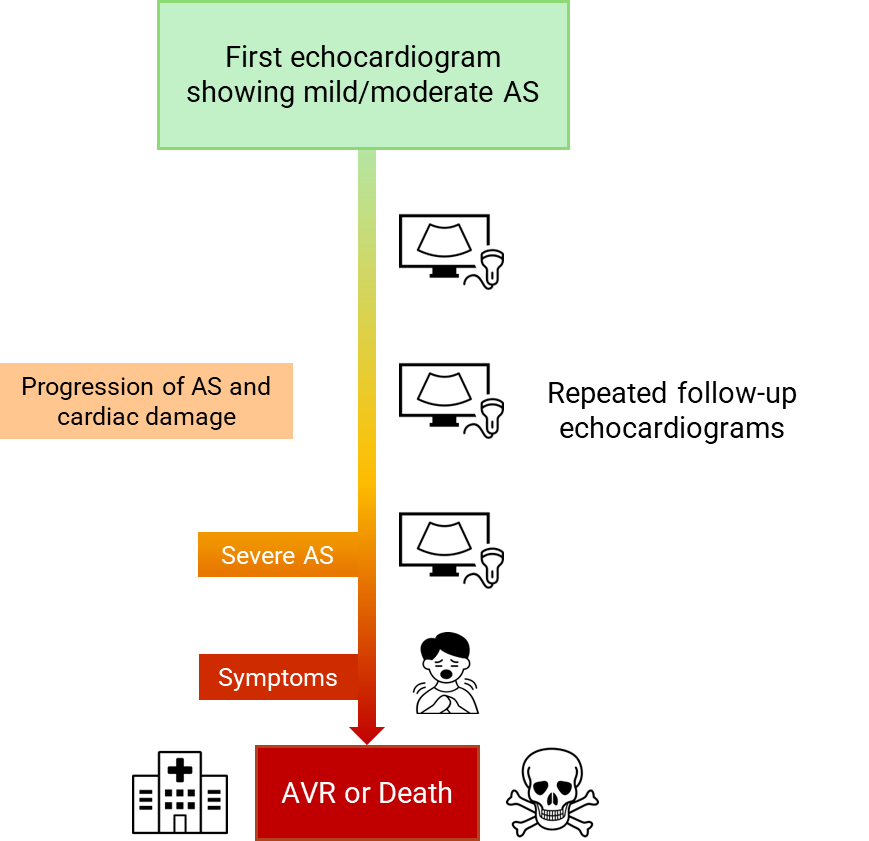


Supplement 3 - Predicted Trajectories of LVMI, RWT, and E/e' With Increasing Mean Gradient


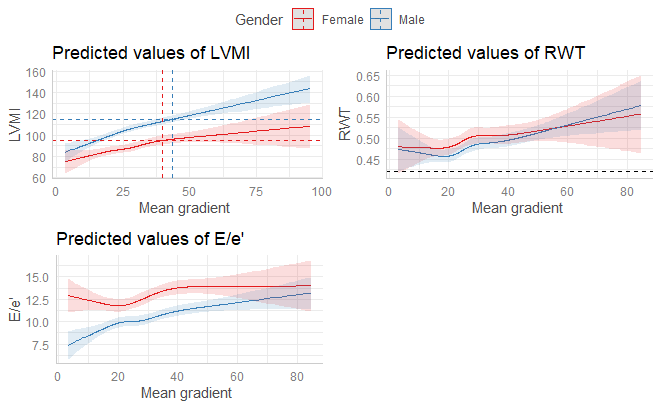


Supplement 4 - Echocardiographic Characteristics at Symptom Development

|  | **All (N=234)** | **Female (N=93)** | **Male (N=141)** | **P** | **Missing** |
| --- | --- | --- | --- | --- | --- |
| Time from measurement to symptoms (days) | 0.00 [0.00;14.0] | 0.00 [0.00;14.0] | 0.00 [0.00;14.0] | 0.546 | 0 (0.0%) |
| Mean gradient | 39.8 [31.5;47.5] | 40.1 [31.0;49.6] | 38.8 [31.9;44.7] | 0.575 | 8 (3.4%) |
| AVAc | 0.92 [0.76;1.05] | 0.83 [0.73;0.98] | 0.94 [0.80;1.10] | 0.001 | 8 (3.4%) |
| AVA indexed to BSA (cm2/m2) | 0.46 [0.38;0.55] | 0.46 [0.40;0.56] | 0.45 [0.38;0.54] | 0.400 | 8 (3.4%) |
| Peak jet velocity | 4.00 (0.59) | 4.00 (0.60) | 4.00 (0.59) | 0.929 | 0 (0.0%) |
| LVMI | 103 [84.0;124] | 92.0 [75.0;108] | 109 [93.4;135] | <0.001 | 10 (4.3%) |
| RWT | 0.47 [0.41;0.57] | 0.51 [0.41;0.58] | 0.46 [0.39;0.56] | 0.171 | 23 (9.8%) |
| Stroke Volume | 83.3 [68.9;97.7] | 77.6 [62.3;87.0] | 88.5 [73.2;108] | <0.001 | 16 (6.8%) |
| E/e' | 10.6 [8.30;15.1] | 11.9 [8.40;16.7] | 10.0 [8.00;14.0] | 0.076 | 76 (32.5%) |
| e' average | 6.85 [5.68;8.83] | 6.30 [4.90;8.75] | 7.20 [6.10;8.90] | 0.027 | 54 (23.1%) |
| Concentric Hypertrophy | 70 (32.1%) | 29 (33.0%) | 41 (31.5%) | 0.943 | 16 (6.8%) |
| Diastolic Dysfunction | 94 (56.3%) | 45 (65.2%) | 49 (50.0%) | 0.073 | 67 (28.6%) |
| Tricuspid Regurgitation Peak Velocity | 2.70 [2.50;3.02] | 2.75 [2.50;3.08] | 2.70 [2.60;3.00] | 0.967 | 446 (82.3%) |

Supplement 5 - Echocardiographic Characteristics Closest to AVR

|  | **All (N=252)** | **Female (N=90)** | **Male (N=162)** | **P** | **Missing** |
| --- | --- | --- | --- | --- | --- |
| Time compared to baseline (years) | 3.82 [2.13;5.75] | 3.41 [2.19;5.64] | 3.93 [2.03;5.79] | 0.958 | 0 (0.0%) |
| Time from measurement to AVR (years) | 0.25 [0.18;0.36] | 0.25 [0.17;0.36] | 0.24 [0.18;0.36] | 0.978 | 0 (0.0%) |
| AVAc | 0.91 [0.76;1.04] | 0.89 [0.73;0.98] | 0.93 [0.79;1.07] | 0.040 | 6 (2.4%) |
| AVA indexed to BSA (cm2/m2) | 0.45 [0.38;0.54] | 0.47 [0.41;0.56] | 0.44 [0.38;0.53] | 0.072 | 6 (2.4%) |
| Peak jet velocity | 4.10 [3.70;4.50] | 4.16 [3.62;4.40] | 4.10 [3.80;4.50] | 0.654 | 0 (0.0%) |
| Mean gradient | 41.0 [33.7;49.3] | 41.4 [32.6;48.5] | 40.8 [34.5;49.3] | 0.778 | 12 (4.8%) |
| Peak gradient | 67.6 [55.3;79.9] | 68.7 [53.2;78.9] | 67.4 [57.0;80.0] | 0.585 | 0 (0.0%) |
| AV VTI | 96.5 (19.3) | 94.6 (21.0) | 97.5 (18.4) | 0.280 | 12 (4.8%) |
| E/e' | 9.80 [7.73;14.3] | 10.7 [8.25;15.2] | 9.50 [7.60;14.0] | 0.169 | 82 (32.5%) |
| e' average | 6.90 [6.00;9.00] | 6.90 [5.50;8.90] | 7.00 [6.07;9.25] | 0.234 | 63 (25.0%) |
| LAVI | 37.9 [30.0;47.0] | 36.0 [29.0;47.0] | 38.5 [30.0;47.8] | 0.240 | 42 (16.7%) |
| LVMI | 106 [86.5;126] | 91.0 [72.0;108] | 112 [98.0;138] | <0.001 | 13 (5.2%) |
| Left Ventricular Hypertrophy (LVH) | 111 (46.4%) | 38 (44.7%) | 73 (47.4%) | 0.791 | 13 (5.2%) |
| LVEDD | 48.0 [43.0;53.0] | 44.0 [40.0;47.0] | 50.0 [46.0;54.0] | <0.001 | 10 (4.0%) |
| LVESD | 31.0 [27.0;37.0] | 28.0 [23.0;32.0] | 33.0 [29.0;39.0] | <0.001 | 18 (7.1%) |
| IVSd | 12.0 [11.0;14.0] | 12.0 [10.0;13.5] | 12.0 [11.0;14.0] | 0.003 | 11 (4.4%) |
| PWd | 10.8 [9.31;12.4] | 9.90 [8.70;11.1] | 11.1 [10.0;12.8] | <0.001 | 11 (4.4%) |
| RWT | 0.49 [0.41;0.57] | 0.51 [0.42;0.58] | 0.48 [0.40;0.56] | 0.288 | 23 (9.1%) |
| Stroke Volume | 86.1 [72.0;99.2] | 78.7 [65.1;87.7] | 89.2 [76.9;106] | <0.001 | 17 (6.7%) |
| Diastolic Dysfunction | 97 (54.8%) | 41 (62.1%) | 56 (50.5%) | 0.176 | 75 (29.8%) |
| Tricuspid Regurgitation Peak Velocity | 2.70 [2.50;3.00] | 2.74 [2.48;3.00] | 2.70 [2.58;3.00] | 0.982 | 446 (82.3%) |

Supplement 6 - Echocardiographic Parameters at Last Echocardiography

|  | **All (N=542)** | **Female (N=205)** | **Male (N=337)** | **P** | **Missing** |  |
| --- | --- | --- | --- | --- | --- | --- |
| Time compared to baseline (years) | 3.93 [2.13;5.94] | 3.95 [2.22;5.85] | 3.93 [2.04;5.98] | 0.729 | 0 (0.0%) | |
| AVAc | 0.97 [0.83;1.18] | 0.93 [0.81;1.13] | 1.00 [0.84;1.21] | 0.014 | 15 (2.8%) | |
| AVAc indexed to BSA | 0.50 [0.42;0.59] | 0.53 [0.44;0.62] | 0.49 [0.40;0.58] | 0.011 | 15 (2.8%) | |
| Peak jet velocity | 3.80 [3.30;4.20] | 3.70 [3.20;4.20] | 3.80 [3.40;4.20] | 0.184 | 0 (0.0%) | |
| Mean gradient | 33.9 [26.6;42.5] | 33.0 [25.7;42.5] | 34.1 [27.1;42.5] | 0.444 | 21 (3.9%) | |
| Peak gradient | 57.1 [44.4;70.7] | 56.0 [42.0;70.4] | 58.0 [46.4;71.0] | 0.165 | 0 (0.0%) | |
| AV VTI | 88.7 (20.1) | 87.3 (20.5) | 89.5 (19.8) | 0.247 | 21 (3.9%) | |
| E/e' | 10.1 [7.70;14.6] | 11.4 [8.40;16.5] | 9.50 [7.50;13.5] | 0.002 | 190 (35.1%) | |
| e' average | 7.20 [5.90;9.00] | 6.90 [5.10;8.82] | 7.40 [6.10;9.20] | 0.006 | 119 (22.0%) | |
| LAVI | 37.0 [28.0;47.2] | 36.5 [29.0;48.0] | 37.3 [28.0;46.8] | 0.882 | 82 (15.1%) | |
| LVMI | 103 [85.1;123] | 92.0 [76.0;109] | 108 [90.8;131] | <0.001 | 31 (5.7%) | |
| Left Ventricular Hypertrophy (LVH) | 225 (44.0%) | 88 (47.1%) | 137 (42.3%) | 0.340 | 31 (5.7%) | |
| LVEDD | 47.0 [43.0;52.0] | 44.0 [40.0;47.0] | 49.0 [45.0;54.0] | <0.001 | 24 (4.4%) | |
| LVESD | 31.0 [26.0;36.7] | 28.0 [23.0;33.0] | 33.0 [28.0;39.0] | <0.001 | 43 (7.9%) | |
| IVSd | 12.0 [11.0;14.0] | 12.0 [10.0;14.0] | 12.0 [11.0;14.0] | 0.010 | 24 (4.4%) | |
| PWd | 10.6 [9.20;12.0] | 10.0 [8.60;11.1] | 11.0 [9.70;12.4] | <0.001 | 27 (5.0%) | |
| RWT | 0.48 [0.40;0.56] | 0.49 [0.41;0.57] | 0.47 [0.40;0.56] | 0.206 | 47 (8.7%) | |
| Stroke Volume | 85.5 [71.8;99.1] | 79.6 [66.6;91.9] | 88.7 [75.0;105] | <0.001 | 39 (7.2%) | |
| Stroke Volume indexed to BSA (ml/m^2^) | 43.4 [36.6;51.0] | 43.3 [36.7;51.2] | 43.5 [36.6;50.9] | 0.803 | 39 (7.2%) | |
| Diastolic Dysfunction | 205 (52.3%) | 90 (60.8%) | 115 (47.1%) | 0.012 | 150 (27.7%) | |
| Tricuspid Regurgitation Peak Velocity | 2.70 [2.50;3.10] | 2.70 [2.50;3.10] | 2.70 [2.50;3.10] | 0.638 | 333 (61.4%) | |

Supplement 7 – Baseline Characteristics Total Population and Missing Values

|  | **Overall (N=542)** | **Missing** |
| --- | --- | --- |
| Total follow-up duration | 6.47 [4.09;8.90] | 0 (0.0%) |
| Age at diagnosis | 69.0 [59.0;76.0] | 0 (0.0%) |
| Current smoker | 171 (31.5%) | 0 (0.0%) |
| BMI (kg/m2) | 27.1 [24.1;30.4] | 0 (0.0%) |
| Bicuspid aortic valve | 92 (17.0%) | 0 (0.0%) |
| Diabetes | 121 (22.3%) | 0 (0.0%) |
| Hypertension | 324 (59.8%) | 0 (0.0%) |
| Coronary artery disease | 120 (22.1%) | 0 (0.0%) |
| Previous myocardial infarction | 57 (10.5%) | 0 (0.0%) |
| Atrial fibrillation | 101 (18.6%) | 0 (0.0%) |
| Heart failure | 40 (7.38%) | 0 (0.0%) |
| Intracardiac device | 29 (5.35%) | 0 (0.0%) |
| Previous CVA | 77 (14.2%) | 0 (0.0%) |
| Peripheral vascular disease | 53 (9.78%) | 0 (0.0%) |
| Chronic kidney disease (eGFR < 60) | 53 (9.78%) | 0 (0.0%) |
| Chronic dialysis | 32 (5.90%) | 0 (0.0%) |
| Chest irradiation | 28 (5.17%) | 0 (0.0%) |
| COPD/asthma | 82 (15.1%) | 0 (0.0%) |
| Beta-blocker | 232 (42.8%) | 0 (0.0%) |
| ACE-inhibitors | 171 (31.5%) | 0 (0.0%) |
| MRA | 24 (4.43%) | 0 (0.0%) |
| Statin | 245 (45Í.2%) | 0 (0.0%) |
| Oral anticoagulant | 99 (18.3%) | 0 (0.0%) |
| NT-proBNP | 331 [147;805] | 424 (78.2%) |
| Hemoglobin (mmol/L) | 8.40 [7.57;9.20] | 90 (16.6%) |
| Sodium (mmol/L) | 140 [138;142] | 78 (14.4%) |
| Potassium (mmol/L) | 4.20 [4.00;4.50] | 78 (14.4%) |
| BUN (mg/dL) | 18.2 [14.3;23.7] | 96 (17.7%) |
| Creatinine (umol/L) | 83.0 [69.0;104] | 72 (13.3%) |
| eGFR (CKD-EPI) | 74.0 [58.0;89.0] | 99 (18.3%) |
| Total cholesterol | 4.50 [3.70;5.40] | 180 (33.2%) |
| LDL-cholesterol | 2.60 [2.00;3.40] | 186 (34.3%) |
| HDL-cholesterol | 1.30 [1.00;1.60] | 193 (35.6%) |
| Triglycerides | 1.58 [1.07;2.24] | 234 (43.2%) |

Supplement 8 – Baseline Echocardiographic Characteristics Total Population and Missing Values

|  | **All (N=542)** | **Missing** |
| --- | --- | --- |
| Baseline AS severity: |  | 0 (0.0%) |
| Mild | 69 (12.7%) |  |
| Moderate | 438 (80.8%) |  |
| Severe | 35 (6.46%) |  |
| AVAc | 1.29 [1.12;1.46] | 22 (4.1%) |
| AVAc indexed to BSA | 0.66 [0.58;0.75] | 22 (4.1%) |
| Peak jet velocity | 3.00 [2.80;3.30] | 0 (0.0%) |
| Mean gradient | 21.2 [17.3;25.5] | 62 (11.4%) |
| LVEF < 50% | 47 (8.67%) | 0 (0.0%) |
| SV index < 35 mL/m^2^ | 71 (14.7%) | 58 (10.7%) |
| EF/Flow category: |  | 58 (10.7%) |
| Low EF, low-flow | 14 (2.89%) |  |
| Low EF, normal-flow | 28 (5.79%) |  |
| Normal EF, low-flow | 57 (11.8%) |  |
| Normal EF, normal-flow | 385 (79.5%) |  |
| RWT | 0.45 [0.39;0.54] | 165 (30.4%) |
| LVMI | 89.0 [73.0;107] | 47 (8.7%) |
| Left Ventricular Hypertrophy (LVH) | 121 (24.4%) | 47 (8.7%) |
| e’ average | 8.00 [6.60;9.90] | 140 (25.8%) |
| E/e’ | 9.45 [7.60;12.3] | 178 (32.8%) |
| E/A | 0.85 [0.69;1.06] | 174 (32.1%) |
| Diastolic Dysfunction (ASE Definition) | 154 (41.4%) | 170 (31.4%) |
| Pulmonary Artery Systolic Pressure | 32.6 [27.9;38.4] | 357 (65.9%) |
| Tricuspid Regurgitation Peak Velocity | 2.6 [2.40;2.90] | 357 (65.9%) |
| Mitral Regurgitation: |  | 3 (0.6%) |
| None-Trace | 405 (75.1%) |  |
| Mild | 103 (19.1%) |  |
| Moderate | 28 (5.19%) |  |
| Severe | 3 (0.56%) |  |
| Aortic Regurgitation: |  | 3 (0.6%) |
| None-Trace | 352 (65.3%) |  |
| Mild | 139 (25.8%) |  |
| Moderate | 44 (8.16%) |  |
| Severe | 4 (0.74%) |  |
| Tricuspid Regurgitation: |  | 7 (1.3%) |
| None-Trace | 405 (75.7%) |  |
| Mild | 104 (19.4%) |  |
| Moderate | 21 (3.93%) |  |
| Severe | 5 (0.93%) |  |

*Supplement 9 - Rates of Change of Echocardiographic Parameters Total Population and Missing Values*

|  | **All (N=542)** | **Missing** |
| --- | --- | --- |
| AVA Slope (change per year) | -0.09 [-0.15;-0.04] | 2 (0.4%) |
| Mean Gradient Slope (change per year) | 3.08 [1.54;5.73] | 3 (0.6%) |
| Peak Velocity Slope (change per year) | 0.17 [0.09;0.30] | 0 (0.0%) |
| Rapid progression | 270 (50.0%) | 2 (0.4%) |
| LVMI Slope (g/m2/year) | 2.83 [-0.14;6.79] | 16 (3.0%) |
| RWT Slope | 0.01 [-0.01;0.02] | 40 (7.4%) |
| e’ Average Slope | -0.16 [-0.47;0.11] | 37 (6.8%) |

*Supplement 10 – Outcomes Total Population and Missing Values*

|  | **All (N=542)** | **Missing** |
| --- | --- | --- |
| Hospitalisation for heart failure | 87 (16.2%) | 4 (0.7%) |
| Number of HF hospitalisations | 1.00 [1.00;2.00] | 4 (0.7%) |
| Death | 186 (34.3%) | 0 (0.0%) |
| Cause of death : |  | 0 (0.0%) |
| Cardiovascular | 44 (23.9%) |  |
| Non-cardiovascular | 74 (40.2%) |  |
| Unknown | 66 (35.9%) |  |
| New-onset heart failure | 62 (12.4%) | 43 (7.9%) |
| New-onset atrial fibrillation | 99 (22.1%) | 94 (17.3%) |
| Myocardial infarction: |  | 0 (0.0%) |
| No | 503 (92.8%) |  |
| NSTEMI | 27 (4.98%) |  |
| STEMI | 12 (2.21%) |  |
| Percutaneous coronary intervention | 56 (10.3%) | 0 (0.0%) |
| CABG | 38 (7.01%) | 0 (0.0%) |
| Cerebrovascular accident | 35 (6.51%) | 4 (0.7%) |
| Peripheral artery disease intervention/surgery | 26 (5.14%) | 36 (6.6%) |
| Device implantation: |  | 87 (16.1%) |
| No | 416 (91.4%) |  |
| Pacemaker | 28 (6.15%) |  |
| ICD | 3 (0.66%) |  |
| CRT | 8 (1.76%) |  |

Sensitivity Analysis

*Supplement 11 – Echocardiographic parameters at the first echocardiography showing moderate aortic stenosis (excluding patients with mild AS, reduced ejection fraction, reduced stroke volume index, history of coronary artery disease, or history of chest irradiation)*

|  | **All (N=315)** | **Female (N=128)** | **Male (N=187)** | **P** |
| --- | --- | --- | --- | --- |
| AVAc | 1.21 [1.12;1.33] | 1.17 [1.10;1.28] | 1.25 [1.13;1.35] | 0.001 |
| AVA indexed to BSA (cm2/m2) | 0.64 [0.58;0.68] | 0.66 [0.59;0.73] | 0.62 [0.57;0.66] | <0.001 |
| Peak jet velocity | 3.30 [3.20;3.58] | 3.30 [3.10;3.50] | 3.40 [3.20;3.60] | 0.005 |
| Mean gradient | 25.4 [22.8;29.6] | 24.4 [22.6;27.4] | 26.4 [23.1;30.6] | 0.005 |
| LVEDD | 46.5 (6.43) | 43.7 (5.18) | 48.4 (6.54) | <0.001 |
| LVESD | 29.6 (6.51) | 27.9 (5.73) | 30.8 (6.77) | <0.001 |
| IVSd | 11.0 [10.0;13.0] | 11.0 [9.00;13.0] | 12.0 [11.0;13.0] | 0.008 |
| PWd | 10.0 [8.75;11.2] | 9.40 [8.25;11.0] | 10.3 [9.17;11.5] | 0.001 |
| LAVI | 32.0 [27.0;39.0] | 35.0 [28.0;41.0] | 31.0 [26.0;37.5] | 0.030 |
| RWT | 0.46 [0.39;0.55] | 0.47 [0.39;0.56] | 0.45 [0.40;0.54] | 0.564 |
| LVMI | 92.0 [75.0;114] | 86.0 [69.0;102] | 100 [80.0;119] | <0.001 |
| Left Ventricular Hypertrophy (LVH) | 93 (31.3%) | 41 (33.3%) | 52 (29.9%) | 0.614 |
| e' average | 8.20 [6.60;10.3] | 8.10 [6.40;10.1] | 8.40 [6.90;10.4] | 0.239 |
| E/e' | 9.20 [7.50;11.8] | 10.2 [8.30;13.9] | 8.60 [6.90;11.1] | <0.001 |
| Mitral Regurgitation: |  |  |  | 0.329 |
| None-Trace | 106 (79.7%) | 40 (74.1%) | 66 (83.5%) |  |
| Mild | 22 (16.5%) | 12 (22.2%) | 10 (12.7%) |  |
| Moderate | 5 (3.76%) | 2 (3.70%) | 3 (3.80%) |  |
| Aortic Regurgitation: |  |  |  | 0.791 |
| None-Trace | 84 (63.2%) | 36 (66.7%) | 48 (60.8%) |  |
| Mild | 37 (27.8%) | 15 (27.8%) | 22 (27.8%) |  |
| Moderate | 11 (8.27%) | 3 (5.56%) | 8 (10.1%) |  |
| Severe | 1 (0.75%) | 0 (0.00%) | 1 (1.27%) |  |
| Tricuspid Regurgitation: |  |  |  | 0.197 |
| None-Trace | 102 (77.9%) | 38 (71.7%) | 64 (82.1%) |  |
| Mild | 27 (20.6%) | 14 (26.4%) | 13 (16.7%) |  |
| Moderate | 1 (0.76%) | 1 (1.89%) | 0 (0.00%) |  |
| Severe | 1 (0.76%) | 0 (0.00%) | 1 (1.28%) |  |

*Supplement 12 – Concentric hypertrophy and diastolic dysfunction by increasing mean gradient curves excluding patients with a rEF or low SVi*


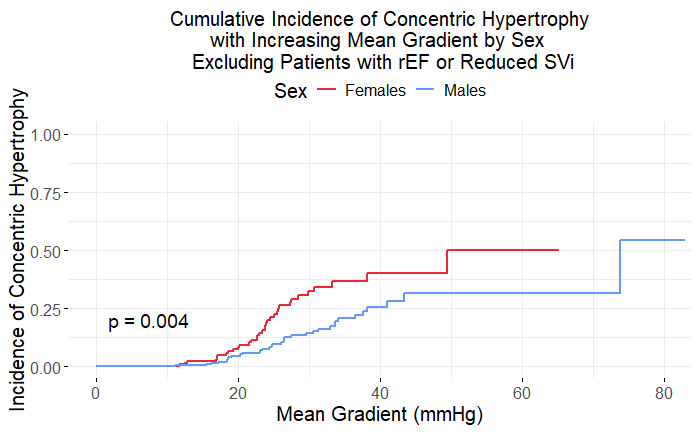

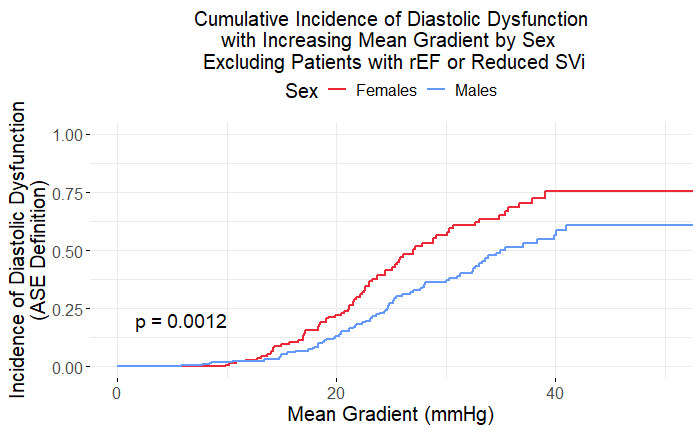


*Supplement 13 – KM Curves using moderate AS baseline*


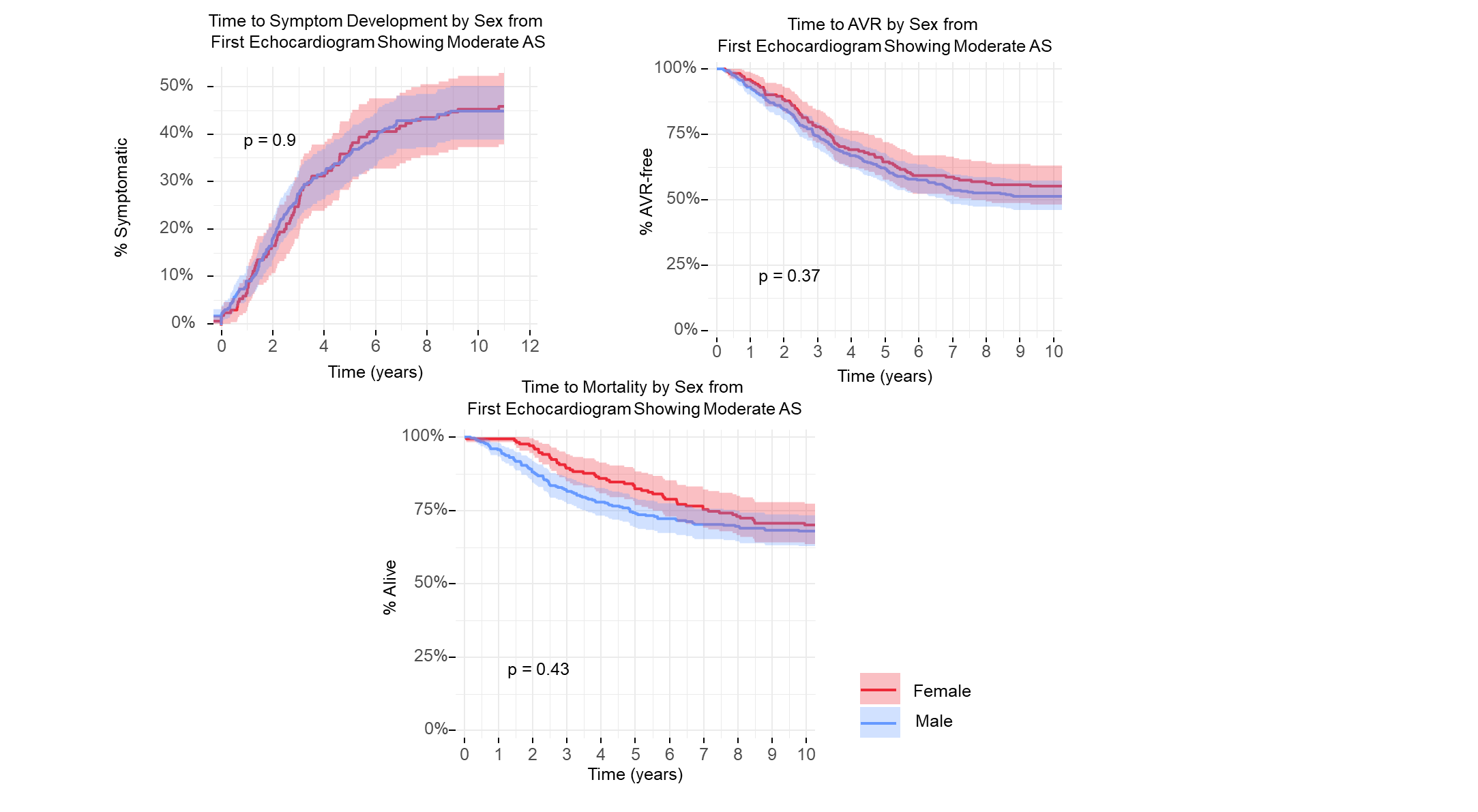

Supplement: qyag064_Supplementary_Data [file qyag064_supplementary_data.docx]
